# Health seeking behavior following snakebites in Sri Lanka: Results of an island wide community based survey

**DOI:** 10.1371/journal.pntd.0006073

**Published:** 2017-11-06

**Authors:** Dileepa Senajith Ediriweera, Anuradhani Kasturiratne, Arunasalam Pathmeswaran, Nipul Kithsiri Gunawardena, Shaluka Francis Jayamanne, David Griffith Lalloo, Hithanadura Janaka de Silva

**Affiliations:** 1 Centre for Health Informatics, Biostatistics and Epidemiology, Faculty of Medicine, University of Kelaniya, Ragama, Sri Lanka; 2 Department of Public Health, Faculty of Medicine, University of Kelaniya, Ragama, Sri Lanka; 3 Department of Parasitology, University of Kelaniya, Ragama, Sri Lanka; 4 Department of Medicine, Faculty of Medicine, University of Kelaniya, Ragama, Sri Lanka; 5 Liverpool School of Tropical Medicine, Liverpool, United Kingdom; Universidad de Costa Rica, COSTA RICA

## Abstract

**Introduction:**

Sri Lanka has a population of 21 million and about 80,000 snakebites occur annually. However, there are limited data on health seeking behavior following bites. We investigated the effects of snakebite and envenoming on health seeking behavior in Sri Lanka.

**Methods:**

In a community-based island-wide survey conducted in Sri Lanka 44,136 households were sampled using a multistage cluster sampling method. An individual who reported experiencing a snakebite within the preceding 12 months was considered a case. An interviewer-administered questionnaire was used to obtain details of the bite and health seeking behavior among cases.

**Results:**

Among 165,665 individuals surveyed, there were 695 snakebite victims. 682 (98.1%) had sought health care after the bite; 381 (54.8%) sought allopathic treatment and 301 (43.3%) sought traditional treatment. 323 (46.5%) had evidence of probable envenoming, among them 227 (70.3%) sought allopathic treatment, 94 (29.1%) sought traditional treatment and 2 did not seek treatment. There was wide geographic variation in the proportion of seeking allopathic treatment from <20% in the Western province to > 90% in the Northern province. Multiple logistic regression analysis showed that seeking allopathic treatment was independently associated with being systemically envenomed (Odds Ratio = 1.99, 95% CI: 1.36–2.90, P < 0.001), distance to the healthcare facility (OR = 1.13 per kilometer, 95% CI: 1.09 to 1.17, P < 0.001), time duration from the bite (OR = 0.49 per day, 95% CI: 0.29–0.74, P = 0.002), and the local incidence of envenoming (OR = 1.31 for each 50 per 100,000, 95% CI: 1.19–1.46, P < 0.001) and snakebite (OR = 0.90 for each 50 per 100,000, 95% CI: 0.85–0.94, P < 0.001) in the relevant geographic area.

**Conclusions:**

In Sri Lanka, both allopathic and traditional treatments are sought following snakebite. The presence of probable envenoming was a major contribution to seeking allopathic treatment.

## Author summary

Sri Lanka has a high incidence of snakebite with 80,000 bites occurring annually in a population of 21 million. However, there are very limited data on health seeking behaviour following bites. We undertook a community based island-wide survey in Sri Lanka during 2012–2013 and surveyed 165,665 individuals living in 44,136 households. There were 695 snakebite victims and 98% of them had sought health care after the bite; 55% sought allopathic (western medical) treatment and 43% sought traditional treatment. The decision on seeking allopathic treatment was influenced mainly by probable envenoming following the snakebite.

## Introduction

Sri Lanka has a very rich snake fauna. There are 96 snake species in the country but only six of them are considered venomous (i.e. *Naja naja*, *Bungarus ceylonicus*, *Bungarus caeruleus*, *Daboia russelii*, *Echis carinatus and Hypnale hypnale)*. The country has a high incidence of bites and envenoming, but a relatively low mortality associated with snakebite. A national survey conducted in 2012 estimated that there are an estimated 80,000 snakebites, 30,000 envenomings and 400 deaths annually (in a total population of about 20 million according to the National Census Data for Sri Lanka—2012) [1]. Both snakes and snakebites have a wide geographical variation [1]. The Russell’s viper is considered as the largest and most dangerous snake in Sri Lanka and is the most widely distributed venomous snake. Hump nosed vipers are commonly found in human habitats including rubber, tea, coconut and cocoa plantations and 35% to 45% of human bites result from this species. Saw scaled vipers are largely confined to the arid dry zones of the country including Northern and Eastern Provinces and are responsible for only 1% to 2% of the total bites. Cobras are the largest elapids found in Sri Lanka and are also widely distributed in the country. Kraits are mainly found in and around human habitats, and the majority of krait bites have been reported from within households in the dry zone of the country. Cobra and krait bites mainly cause neurotoxic effects while viper bites are principally associated with haematological abnormalities and soft tissue damage. Sri Lankan Russell’s viper bites could also cause neurotoxicity [2–4].

State healthcare is free at the point of delivery in Sri Lanka and includes both allopathic and indigenous medical systems. The state healthcare sector has a total of 17,000 allopathic practitioners (MBBS qualified doctors) and over 20,000 registered indigenous practitioners (84.6% Ayurveda, 12.7% Siddha system and 2.7% other). It is estimated that there are another nearly 8,000 who are descendants of traditional medical practitioners who also provide healthcare [5,6].

In many rural communities in low and middle income countries, including Sri Lanka, victims traditionally favour indigenous treatments for snakebites. In rural Bangladesh, Nigeria and Kenya, only 3%, 8.5% and 27% of snakebite victims respectively, sought hospital treatment [7–9]. This pattern of health seeking behaviour may be due to a combination of socio-cultural factors and poor access to often sub-optimal health care in these countries. Two qualitative studies investigating beliefs regarding snakebite and their influence on health seeking behaviour in four rural communities in Sri Lanka found that people firmly believed that Ayurvedic treatment was effective for snakebite [9,10]. Traditional healers were respected and although many victims finally sought allopathic treatment, they often initially consulted a traditional practitioner [10]. There were also common misconceptions about harms of hospital treatment, based mainly on misinformation regarding antivenom therapy [11]. However, in some middle income countries, such as Costa Rica, most of the population affected by snakebites seeks treatment in health posts where free allopathic treatment is provided [12].

Outcomes of snakebites could be associated with the health seeking behavior of victims following the bite, and the decision on health seeking behavior can be influenced by the victims’ social and natural environment [1,13]. Information on health seeking behavior from hospital based studies is bound to be biased and cannot be generalized to a community [14,15]. We report health seeking behavior following snakebite and factors influencing the decisions regarding health seeking among the individuals bitten by snakes as part of a community-based island-wide survey on snakebite in Sri Lanka.

## Methods

An island wide community based “National Snakebite Survey” was conducted from 2012 to 2013 to collect data on snakebite [1]. The study was designed to sample about 1% of the Sri Lankan population. A Grama Niladhari division, which is the smallest administrative division in the country, was considered a cluster in the survey methodology. Sri Lanka has nine provinces and these nine provinces are further subdivided to 25 districts (i.e. the number of districts varies from 2 to 5 within provinces). The sample was equally distributed among the nine provinces where 125 Grama Niladhari divisions (clusters) were sampled from each province. These 125 clusters were proportionally allocated to the districts within the province based on the population living in the districts. Of the total of 14,022 GN divisions in Sri Lanka, 1125 GN divisions (125 from each of the 9 provinces) were allocated for the survey. In each cluster, 40 consecutive households were sampled based on a random starting point and all the permanent members of the households were included in the study. An interviewer administered questionnaire was used to collect data where direct interviews were done from an adult household member. Demographic data on household members and snakebite events which occurred within the previous year were recorded. Detailed data on snakebite and health seeking behavior following the snakebite were collected, this included place of bite, time of the bite, identification of snake, primary treatment modality, other treatment modalities if secondary healthcare sought, nature of treatment taken–whether inpatient or outpatient, state sector or private sector care, distance to health care facility, time taken to seek treatment, clinical features and outcome of bite. The presence of probable envenoming in the victims was determined based on the reports of presence of local tissue necrosis at the site of bite, presence of neurotoxicity or bleeding manifestations.

The proportion of victims who sought allopathic and traditional treatment within each province was evaluated. Health seeking behavior was compared between provinces using Western Province, which had the lowest proportion of people seeking allopathic treatment, as the comparator (i.e. the reference level). Linear logistic models were used to model the individual health seeking behavior (i.e. allopathic vs traditional) in relation to the corresponding province, considering the province as the independent variable. Odds ratios with 95% Confidence Intervals were generated to illustrate the differences in health seeking behavior each province.

Estimated snakebite and envenoming bite incidences at the *Grama Niladhari* (GN) divisions (cluster level for this study) were used for the analysis. Each individual snakebite recorded in the national survey and the health seeking behavior activity following that bite was presented graphically with respect to the relevant GN snakebite and envenoming incidences using scatter plots.

There were 695 snakebites in the survey sample and 13 had not sought treatment. Therefore, 682 victims’ data was used for the model. Health seeking behaviour (i.e. allopathic treatment seekers vs indigenous treatment seekers) was considered as the response variable, where those who sought allopathic treatment were coded as 1 and others were coded as 0. Separate binomial logistic models were used to evaluate the differences in health seeking behavior in different provinces, and to identify the variables associated with individual decisions on selecting the treatment type. In the first binomial logistic model, province was considered as an independent variable, where allopathic treatment seeking behavior was compared between provinces. Western Province, which had the lowest proportion of people seeking allopathic treatment was considered as the comparator (i.e. the reference level). Odds ratios with 95% Confidence Intervals were generated to illustrate the differences in health seeking behavior in each province with reference to Western Province. A second binomial logistic model was used to investigate the association between health seeking behavior (i.e. allopathic vs traditional) and socio-demographic variables of victim, envenoming status of victim, time of bite, month of bite, place of bite, identification of snake, distance from the bite location to the healthcare facility where the initial treatment was obtained, outcome of the bite and incidence of snakebite or envenoming at the geographical cluster level. Adjusted Odds ratios with 95% Confidence Intervals for the significant variables were obtained and a p value of 0.05 was considered as significant. Data analysis was done with R programming language version 3.2.3 and geographical mapping was done with ArcMap 10.3.1.

Ethical approval for the study was obtained from the Ethics Review Committee of the Faculty of Medicine, University of Kelaniya. All interviews were conducted after obtaining informed written consent. Approval from District and Divisional level public administrators were obtained for conducting the community-based survey. No animals were used in the study.

## Results

The “National Snakebite Survey” included 165,665 individuals in Sri Lanka. 695 individuals had experienced a snakebite event during the preceding year. Most snakebites (85.8%) had occurred outdoors and 75.4% patients had recognized the offending snake. 323 (46.5%) patients had clinical features of probable envenoming; 92.7% of patients sought treatment within the first day (24 hours) and another 5.0% during the second day.

Patterns of health seeking behavior following the bite are detailed in Table 1. 13 of 695 (1.9%) victims did not seek health care. 381 (54.8%) snakebite victims sought allopathic treatment initially and 301 (43.3%) snakebites sought traditional treatment initially. Secondary treatment was sought by 115 victims (16.7%) after primary treatment—some changed modality but others sought further advice within the same health care system. 24 (64.9%) out of 37 who previously sought indigenous treatment and 39 (50%) out of 78 who previously sought indigenous changed from their initial treatment to allopathic and indigenous healthcare respectively. However, in approximately 30% of these who accessed treatment from a different source within the first 24 hours, 24 (70.6%) of these were envenomed and approximately three quarters of these sought allopathic treatment.

**Table 1:** Pattern of health seeking behaviour following snakebite.

		Primary heath seeking behaviour		Secondary health seeking behaviour	
		Allopathic treatment	Traditional treatment	Allopathic treatment	Traditional treatment
Envenomed	(n = 321)	227 (70.7%)	94 (29.2%)	47 (14.6%)	37 (11.5%)
Non-envenomed	(n = 361)	154 (42,7%)	207 (57.3%)	16 (4.4%)	15 (4.1%)
Inpatient care	(n = 369)	346 (93.8%)	23 (6.2%)	Not available	Not available

All the percentages are calculated using row totals (i.e. numbers given in the first column). Inpatient care includes both envenomed and non-envenomed individuals. Secondary health seeking denotes number of individuals from those who had sought primary health seeking initially.

The time of the bite did not influence whether allopathic or traditional treatment was sought. 138 (19.9%) victims received religious blessings following the snakebite; this proportion did not differ between those seeking allopathic or traditional treatment (19.2% vs 21.7% respectively).

Table 2 demonstrates the wide geographical differences in the pattern of health seeking behavior. More than 85% sought allopathic treatment in Northern and North central provinces in which high proportions of victims are envenomed (71.2% and 70.6% respectively) and which have the highest envenoming incidence among the provinces. Allopathic treatment seeking was lowest in the Western province which has the lowest proportion of bites that lead to envenoming and lowest incidence of envenoming in the country [1].

**Table 2:** Country profile versus health seeking behavior.

Province	Estimated snakebite incidence (per 100,000)	Estimated incidence of envenoming (per 100,000)	Number of government hospitals	Number of bites in sample	Number of envenomed patients in sample	Number of patients seeking allopathic treatment
Western	325 (217–432)	64 (027–101)	150	61	12 (19.7%)	11 (18.0%)
Sabaragamuwa	548 (438–658)	188 (127–249)	94	102	35 (34.3%)	19 (18.6%)
Central	277 (182–371)	92 (042–142)	168	45	15 (33.3%)	15 (33.3%)
Southern	461 (338–584)	95 (047–144)	125	87	18 (20.7%)	42 (48.3%)
Uva	328 (242–414)	203 (137–270)	93	63	39 (61.9%)	33 (52.4%)
North Western	499 (392–605)	184 (118–251)	148	92	34 (37.0%)	62 (67.4%)
Eastern	368 (227–509)	242 (119–365)	131	67	44 (65.7%)	44 (65.7%)
North central	623 (487–760)	440 (325–555)	88	119	84 (70.6%)	100 (84.0%)
Northern	324 (219–428)	230 (145–316)	102	59	42 (71.2%)	55 (93.2%)
Sri Lanka	398 (356–441)	151 (130–173)	1099	695	323 (46.5%)	381 (54.8%)

Data source—Ediriweera DS, et al. (2016) Mapping the Risk of Snakebite in Sri Lanka—A National Survey with Geospatial Analysis. PLoS Negl Trop Dis 10(7): e0004813. https://doi.org/10.1371/journal.pntd.0004813

Allopathic health care seeking was least common in Western province and this province was therefore used as the comparator. There was no significant difference between patterns of health care seeking between Western province and Central and Sabaragamuwa provinces. All other provinces showed significantly higher odds ratios for seeking allopathic treatment which varied from 4.24 in Southern Province to 62.5 in Northern Province (Table 3).

**Table 3:** Allopathic treatment seeking following snakebite in individual provinces compared to Western province.

Province name	Parameter Estimate	Standard Error	Odds ratio	95% Confidence Interval
Intercept	-1.51	0.33	-	-
Sabaragamuwa	0.04	0.42	1.04	0.46–2.42
Central	0.82	0.46	2.27	0.93–5.71
Southern	1.45	0.40	4.24	2.00–9.58
Uva	1.61	0.42	5.00	2.26–11.74
Eastern	2.16	0.42	8.70	3.92–20.61
North Western	2.24	0.40	9.39	4.42–21.43
North Central	3.17	0.42	23.92	10.95–56.53
Northern	4.14	0.62	62.50	20.68–241.13

**Fig 1:**
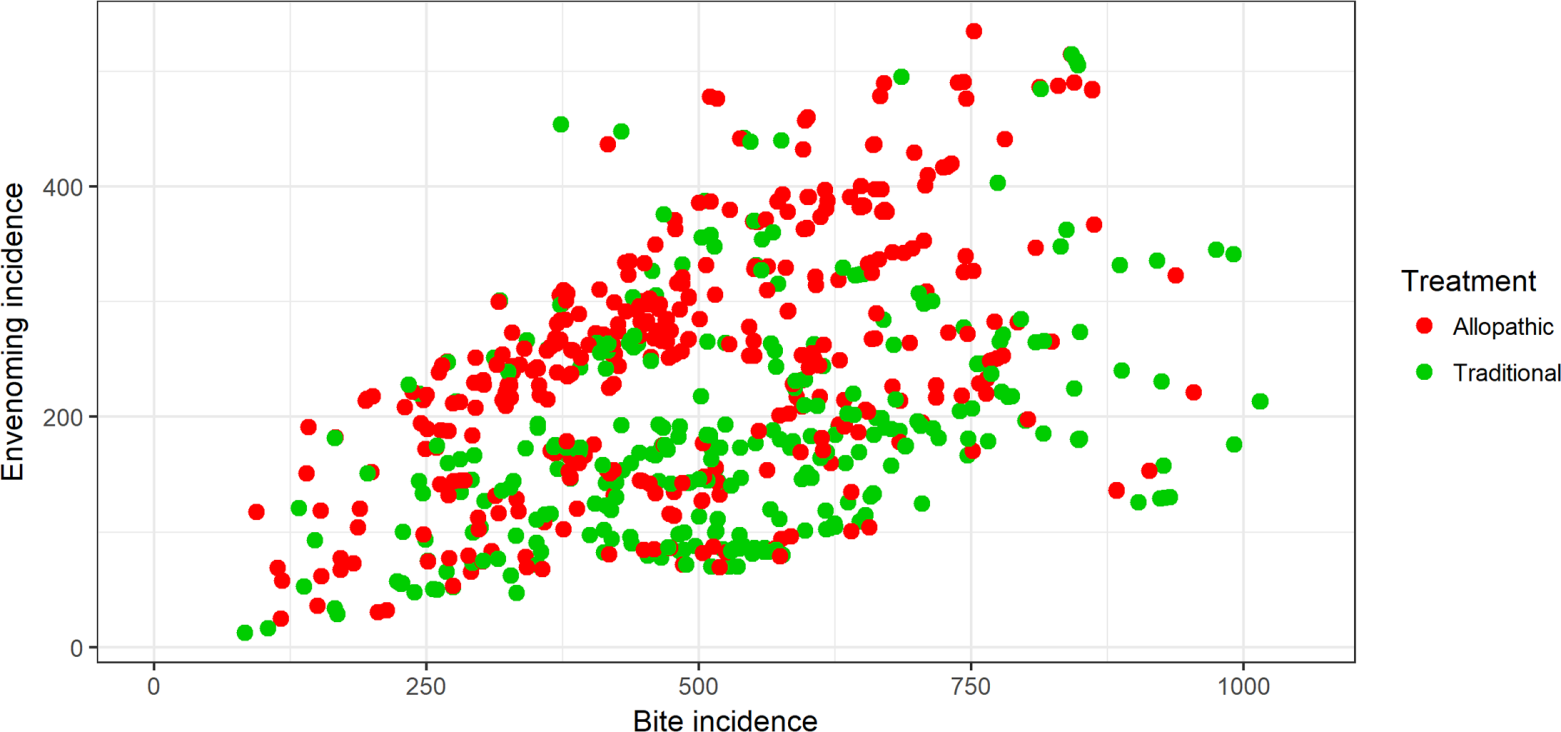
Health seeking behaviour: Snakebite incidence vs envenoming bite incidence. Health seeking behavior pattern with respect to snakebite and envenoming bite incidences of the country.

**Fig 2:**
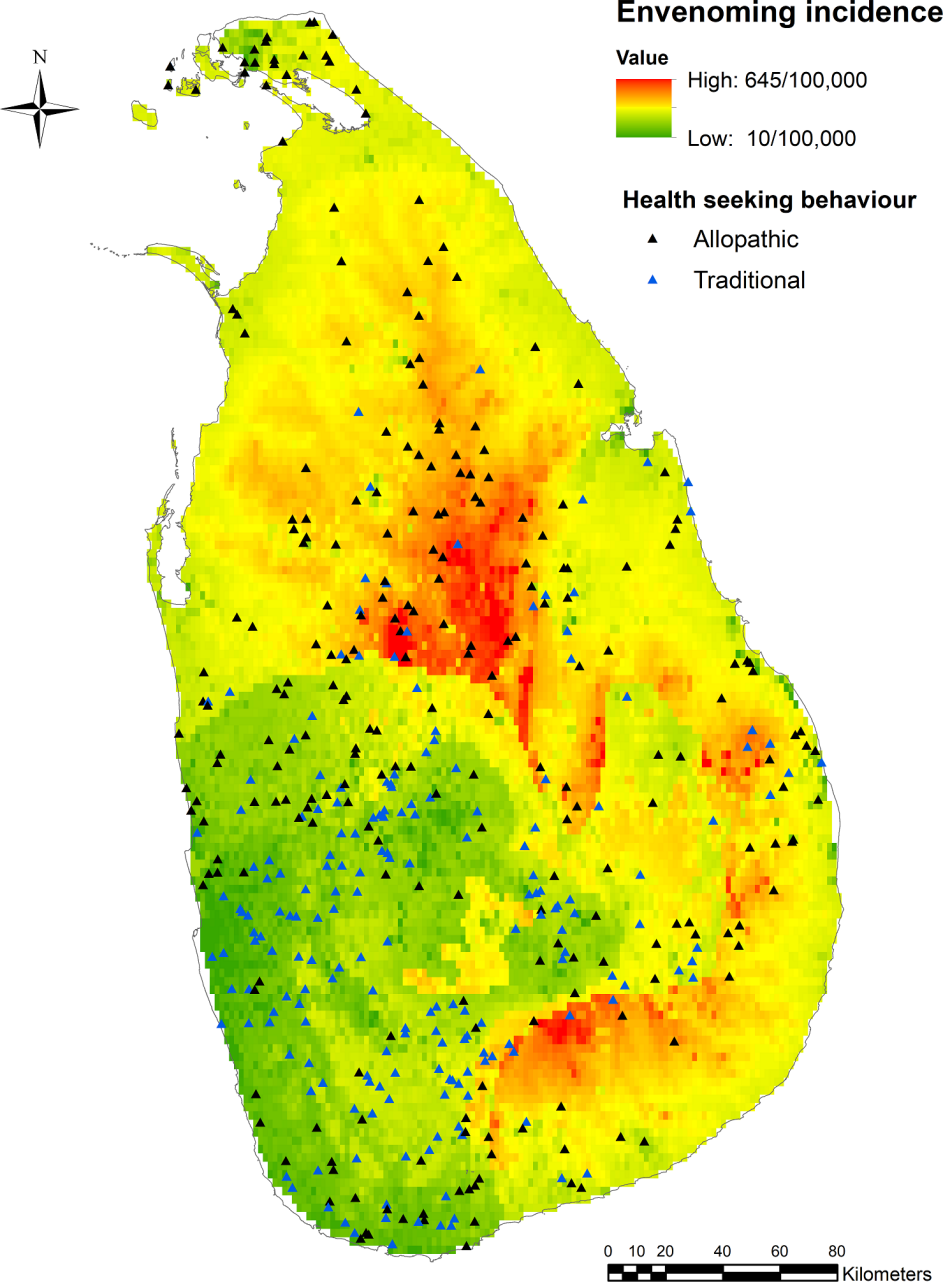
Health seeking behaviour pattern versus envenoming incidence in Sri Lanka. Individual cases are mapped in an envenoming bite incidence map of Sri Lanka. Black trangles shows allopathic treatment seeking behavior and blue trangle shows the traditional treatment seeking behaviour.

The median snake bite incidence was 504 (minimum: 83, inter quartile range: 378–642, maximum: 1511) per 100,000 and the envenoming incidence was 215 (minimum: 13, IQR: 143–283, maximum: 535) per 100,000. Fig 1 shows graphically each bite related to the local incidence of snakebite and envenoming and whether allopathic or traditional health care was sought. Generally, there was greater allopathic healthcare seeking where the incidence of envenoming was high and traditional healthcare seeking was more common where the incidence of envenoming was low (Fig 2).

Multiple logistic regression model fitting showed that health seeking behavior was independently associated with the probable envenoming status of the patient, number of days taken to seek treatment after the bite, distance to the healthcare facility and the envenoming and snakebite incidence in the geographic area. Allopathic treatment seeking was higher in the geographical areas with higher envenoming incidence and among victims with probable envenoming; victims travelled further to the healthcare facility to seek allopathic treatment. On the other hand, allopathic treatment seeking was less common in geographical areas with higher snakebite incidence and in victims who had delayed presentation to healthcare (Table 4).

**Table 4:** Adjusted odds ratios with 95% confidence interval for exposure variables associated with allopathic treatment seeking behavior.

Exposure variable	Parameter Estimate	Standard Error	Odds Ratio	95% Confidence Interval
Intercept	-0.01	0.38	-	-
Probable envenomation	0.69	0.19	1.99	1.36–2.90
Number of days after bite to treatment (per day)	-0.71	0.24	0.49	0.29–0.74
Distance from bite to treatment facility (per km)	0.12	0.02	1.13	1.09–1.17
Snakebite incidence at respective cluster	-0.11	0.02	0.90	0.85–0.94
Envenoming bite incidence at respective cluster	0.27	0.03	1.31	1.19–1.46

Odds ratios for snakebite and envenoming bite incidence are given for an increment of 50 bites per 100,000 population.

## Discussion

Most victims (98.1%) sought some form of treatment following snakebite. Overall, 55% of victims expressed an initial preference for allopathic treatment but this proportion varied considerably across the country. Allopathic treatment seeking behavior was mainly determined by the presence of probable envenoming; 70% of victims with probable envenoming sought allopathic treatment. The incidence of envenoming in a given geographical area was also a significant independent determinant of health seeking behaviour, with an association between increasing incidence of envenoming and allopathic treatment. In contrast, there was an inverse association between the overall incidence of bites in an area and seeking allopathic treatment. This may be related to the fact that geographical areas with the highest incidence of snakebite incidences were not the areas with the highest incidences of envenoming, and many victims with non-envenoming bites tended to seek traditional treatment; familiarity with snakebites may also reduce allopathic health seeking. We did not find any association between health seeking behavior and the victims’ age, gender, ethnicity, religion, employment, income level, education level, month of bite, time of bite, place of bite and type of snake.

More than 90% of snakebite victims sought treatment within the first day. Those who delayed treatment seeking were more likely to seek traditional treatment, probably because these victims experienced lesser degrees of envenoming or no envenoming. Distance from the site of bite to the healthcare facility was also a significant determinant of health seeking behavior; victims who sought allopathic treatment travelled longer distances as previously shown where distance to healthcare facility has impact on individual behaviour [16]. This is likely to simply reflect the easier availability of traditional treatment close to where patients are bitten, but may also be related to those who considered they needed allopathic treatment for more severe manifestations were prepared to travel further.

There are likely many differences in the manner in which people respond to a snakebite. A community based study in Sindh, Pakistan showed that less than 75% of snakebite victims sought any form of healthcare and managed the bites by themselves [17]. In contrast, we found that almost all victims (i.e. 98%) sought some form of treatment. This may be related to high rates of literacy and access to healthcare in Sri Lanka [17]. Another community based study from Kenya showed that 68% of snakebite victims sought treatment from traditional healers, and nearly all victims (98%) initially used traditional treatments [8]. In South Africa, 80% initially used traditional treatments with 63% subsequently accessing a traditional healer [18]. These figures are much higher than in our study [8,18]. The same Kenyan study reported that 25% of bite victims received some form of first aid, including traditional treatment, before reaching a health facility. We found that almost 65% of victims who initially sought traditional treatment subsequently went on to seek allopathic treatment, in line with findings from a previous Sri Lankan hospital based study [15]. Reports from Costa Rica show that most victims seek allopathic treatment soon after a snakebite [19].

Seeking traditional treatment for snakebites has been previously associated with poor access to hospitals [18]. We however found that traditional treatment was often preferred even in the relatively developed areas of the country with good access to hospital care, for example, the Western Province and Central Province are well developed regions in the country and have good access to hospital care with a high concentration of hospitals including teaching hospitals. The Sabaragamuwa Province has both developed and underdeveloped areas and has a Provincial General Hospital and several District Hospitals [18], but the majority of snakebite victims in areas with a high incidence of envenoming sought allopathic treatment, even though these were underserved areas with relatively poor access to hospital care. There were also interesting findings about the time to seek care; in general, allopathic treatment was sought more rapidly than traditional treatment, although people had to travel further to access it. This may reflect the recognition of the urgency when severe snakebite occurs.

Overall, our study reflects the complex decision-making process about health care seeking following snakebite with decisions appearing to be influenced by the community experience of snakebite, as well as the individual circumstances of a bite. The influence of the strength of cultural beliefs is further emphasized by our finding that about one fifth of victims engaged in religious rituals following snakebites and this proportion was similar in those seeking allopathic or traditional treatments.

There are several limitations in our study. We obtained data on snakebite for the preceding one year and this could be associated with recall bias, although an event such as a snakebite is likely to be memorable. The presence or absence of envenoming following snakebite was based on the clinical features reported by the victims, and there is a possibility of misclassification. Distance to the healthcare facility was estimated by patients and so may not be completely accurate. Our analysis was mainly focused on comparing allopathic versus traditional treatment seeking behavior on a national level, and we therefore considered all the non-allopathic treatments as traditional. Individual and community qualitative assessment would have been helpful to generate a more detailed in depth understanding of the personal and household health seeking behaviour following snakebite. Future studies could also be done to assess the effect of macro-ecological variables on health seeking behaviour of snakebite victims in addition to the individual level socio-demographic variables [20].

Nevertheless, we have been able to generate an in-depth analysis of health seeking following snake bite in Sri Lanka, demonstrating that allopathic and traditional treatments are more or less equally sought following snakebite and that although the presence or absence of envenoming are important, many other factors also influence health seeking decisions.

## Supporting information

S1 Dataset Data. (CSV)
